# Hemodynamic Characteristic Analysis of Aneurysm Wall Enhancement in Unruptured Middle Cerebral Artery Aneurysm

**DOI:** 10.3389/fneur.2022.781240

**Published:** 2022-05-09

**Authors:** Weiying Zhong, Yiming Du, Hong Kuang, Ming Liu, Feng Xue, Xue Bai, Donghai Wang, Wandong Su, Yunyan Wang

**Affiliations:** ^1^Department of Neurosurgery, Qilu Hospital of Shandong University and Institute of Brain and Brain-Inspired Science, Shandong University, Jinan, China; ^2^Key Laboratory of Cardiovascular Remodeling and Function Research, Chinese Ministry of Education, Chinese Ministry of Health and Chinese Academy of Medical Sciences, Qilu Hospital of Shandong University, Jinan, China; ^3^State Key Laboratory of Generic Manufacture Technology of Traditional Chinese Medicine, Lunan Pharmaceutical Group Co. Ltd., Linyi, China; ^4^Department of Pharmacy, Yinan County People's Hospital, Linyi, China; ^5^Department of Neurosurgery, The Second Affiliated Hospital of Guangxi Medical University, Nanning, China; ^6^Department of Radiology, Qilu Hospital of Shandong University, Jinan, China

**Keywords:** computational fluid dynamics, magnetic resonance imaging, hemodynamic, intracerebral aneurysm, wall shear stress

## Abstract

**Background and Purpose:**

Aneurysm wall enhancement (AWE) on vessel wall magnetic resonance imaging has been suggested as a marker of the unstable status of intracranial aneurysm (IA) and may predict IA rupture risk. However, the role of abnormal hemodynamics in unruptured IAs with AWE remains poorly understood. This study aimed to determine the association between abnormal hemodynamics and AWE in unruptured middle cerebral artery (MCA) aneurysms.

**Methods:**

A total of 28 patients with 32 bifurcation aneurysms of the middle cerebral artery>3mm in size were retrospectively selected for this study. Vessel wall magnetic resonance images were reviewed, and the AWE pattern of each aneurysm was classified as no AWE, partial AWE, and circumferential AWE. Computational fluid dynamics were used to calculate the hemodynamic variables of each aneurysm. Univariate and multivariate analyses investigated the association between AWE and hemodynamic variables.

**Results:**

AWE was present in 13 aneurysms (40.6%), with 7 (21.9%) showing partial AWE and 6 (18.7%) showing circumferential AWE. Kruskal–Wallis H analysis revealed that hemodynamic variables including wall shear stress (WSS), oscillatory shear index, aneurysm pressure (AP), relative residence time, and low shear area (LSA) were significantly associated with AWE (*p* < 0.05). Further ordinal logistic regression analysis found that WSS was the only factor with a significant association with AWE (*p* = 0.048); similar trends were identified for LSA (*p* = 0.055) and AP (*p* = 0.058). Spearman's correlation analysis showed that AWE was negatively correlated with WSS (rs = −0.622, *p* < 0.001) and AP (rs = −0.535, *p* = 0.002) but positively correlated with LSA (rs = 0.774, *p* < 0.001).

**Conclusion:**

Low wall shear stress, low aneurysm pressure, and increased low shear area were associated with aneurysm wall enhancement on vessel wall magnetic resonance imaging in unruptured cerebral aneurysms. These abnormal hemodynamic parameters may induce inflammation and cause aneurysm wall enhancement. However, the association between these parameters and their underlying pathological mechanisms requires further investigation.

## Introduction

Intracranial aneurysm (IA) is a pathological dilatation of the arterial wall, and its rupture is a major cause of subarachnoid hemorrhage, increasing the risk of high mortality and morbidity. In recent years, more unruptured IAs have been discovered and treated (1). Although not all unruptured IAs rupture during the individual's lifetime, early detection of rupture-prone aneurysms is crucial. There has been an increased focus on developing computational fluid dynamic (CFD) models to investigate the hemodynamics of IAs as growing evidence suggests that hemodynamic factors are an important causative factor for aneurysms, specifically high wall shear stress (WSS) (2, 3). Further studies have reported that low WSS and high oscillatory shear index (OSI) may lead to aneurysm growth and rupture, although these results were not always consistent (4–6).

Inflammation is an important factor for aneurysm remodeling and results in aneurysm growth and rupture (7). Recently, aneurysm wall enhancement (AWE) on vessel wall magnetic resonance imaging (MRI) has been suggested as an indirect marker of aneurysm wall inflammation (8–10). Although the predictive value of AWE for an unstable IA remains uncertain, AWE has been frequently observed in symptomatic aneurysms and aneurysms with growth or rupture during follow-up (11, 12). Therefore, AWE may be an easily available imaging marker reflecting the pathological features of IA and identifying the unstable status of an IA.

Few studies have evaluated the association between aneurysm hemodynamics and AWE on vessel wall MRI (13–19). Some studies reported that low WSS correlated with the AWE region, although one study found no association between hemodynamic parameters and AWE (19). However, these studies often included few cases. Furthermore, the location of the aneurysm influences the hemodynamic variables within aneurysms; for instance, bifurcation and sidewall aneurysms have different hemodynamic parameters (20). Moreover, the morphology of the aneurysm changes after its rupture, and the hemodynamic parameters within aneurysms change after its rupture. Therefore, including cases of ruptured aneurysms would also affect the results of the associations between hemodynamic parameters and AWE (13). A recent study used 4D-flow MRI rather than the CFD model to analyze hemodynamic parameters (18) as 4D-flow MRI may show different hemodynamic results than the CFD analysis (21). Furthermore, the location of the aneurysm may also be associated with AWE, and ruptured aneurysms may have different pathological mechanisms for AWE (22). Therefore, the association between aneurysm hemodynamics and AWE requires further investigation. This study assessed the association between hemodynamic variables and AWE to elucidate the interaction between abnormal hemodynamics and AWE. To eliminate the influence of the factors potentially affecting hemodynamic parameters, only unruptured saccular bifurcation aneurysms of the middle cerebral artery (MCA)>3 mm in size were included in this study, and the CFD model was used to investigate the hemodynamic variables of every aneurysm.

## Materials and Methods

### Patients

This study was approved by the ethics committee of the Qilu Hospital of Shandong University. The requirement of informed consent for this retrospective study was waived. Between May 2019 and August 2021, data from patients with unruptured saccular bifurcation aneurysms of the MCA who underwent preoperative vessel wall MRI were collected retrospectively. All aneurysms had been confirmed by computed tomography angiography or digital subtraction angiography (DSA). The exclusion criteria were as follows: 1) IAs at locations other than at MCA; 2) other types of IAs, such as ruptured, dissecting, or fusiform; 3) aneurysm size <3 mm; 4) concomitant vascular diseases that may influence hemodynamics, such as vascular malformation, severe internal carotid artery, or cerebrovascular stenosis and occlusion; 5) recent administration of aspirin, statins, or non-steroidal anti-inflammatory drugs that may suppress arterial wall enhancement; 6) poor or incomplete MRI, computed tomography angiography, or 3D-DSA imaging data for analysis; and 7) a time interval of over 30 days between DSA or CTA scan for CFD analysis and 3T MRI scan. A total of 28 patients with 32 bifurcation aneurysms of the MCA were included in this study. For the purposes of this study, hypertension was defined as patients treated with antihypertensive medication or patients with a systolic blood pressure ≥140 mm Hg or diastolic blood pressure ≥90 mm Hg. Diabetes mellitus was defined as patients treated with antidiabetic medication or with a fasting plasma glucose level of ≥126 mg/dL, a random plasma glucose level of >200 mg/dL, or a hemoglobin A1c level of ≥6.5%. Hyperlipidemia was defined as patients treated with lipid-lowering drugs, a fasting plasma cholesterol level of ≥6 mmol/L, a fasting plasma triglyceride level of ≥2 mmol/L, or a fasting plasma low-density lipoprotein level of ≥3.5 mmol/L. Current smoking was defined as patients who smoked five cigarettes per day, and current drinking was defined as patients who drank 150 g of alcohol or more per week. Aneurysm size was calculated as the maximum measurement from the neck to the dome on computed tomography angiography or DSA. An irregular aneurysm was defined as a saccular aneurysm with a lobular or daughter sac.

### Computational Fluid Dynamic Analysis

Hemodynamic analysis was performed using a finite element method. Each 3D aneurysm geometric model was generated from computed tomography angiography or 3D digital subtraction angiography DICOM data using Mimics Medical 21 (Materialize, Belgium). Then, the generated aneurysm model was smoothed and trimmed using 3-matic Medical 11(Materialize, Belgium). The generated aneurysm model was then imported and meshed using STAR-CCM+12 (Siemens, German) to create more than four million polyhedral elements with a maximum mesh size of 0.1 mm and four layers of wall prism elements for accurate boundary layer resolution. This study used the Navier-Stokes equations with the assumption of laminar and incompressible flow to evaluate the pulsatile hemodynamics. The blood vessel wall was assumed to be rigid with no-slip boundary conditions. The blood flow was considered a Newtonian fluid with a density of 1,060 kg/m^3^ and a dynamics viscosity of 0.004 Ns/m^2^. The inflow boundary condition was set as a typical pulsatile velocity profile obtained from a healthy person by transcranial Doppler, and the outlet was defined as an opening boundary condition with zero static pressure. Three cardiac cycles were simulated to ensure numeric stability. Each cardiac cycle was set with 1000 time steps for numeric simulation. Also, the simulation results from the last cycle were taken to calculate hemodynamic parameters. The CFD-Post 19.2 (ANSYS, USA) was used to process the calculation of the hemodynamic parameters.

Several hemodynamic parameters were calculated in this study: wall shear stress (WSS), aneurysm pressure (AP), low shear area (LSA), oscillatory shear index (OSI), and relative residence time (RRT) (15, 23). The data presented in this study were time-averaged over the last cycle of flow simulation. Wall shear stress was referred to as the tangential frictional stress caused by the action of blood flow on the vessel wall. Aneurysm pressure and wall shear stress were first time-averaged over the cardiac cycle and then normalized to allow comparison between different patients. Thus, WSS, evaluated in this study, was defined as the time-averaged WSS of an aneurysm wall and then normalized by the average WSS of the parent vessel in the same patient; AP was defined as the time-averaged static pressure normalized by the parent vessel pressure in one cardiac cycle; LSA was defined as the areas of the aneurysm wall exposed to a WSS below 10% of the mean parent arterial WSS and then normalized by the dome area; OSI was defined as the average of the directional changes of WSS over one cardiac cycle; and RRT was defined as the average relative time that blood spends at the wall.

### Vessel Wall MRI Scan

**Figure 1:**
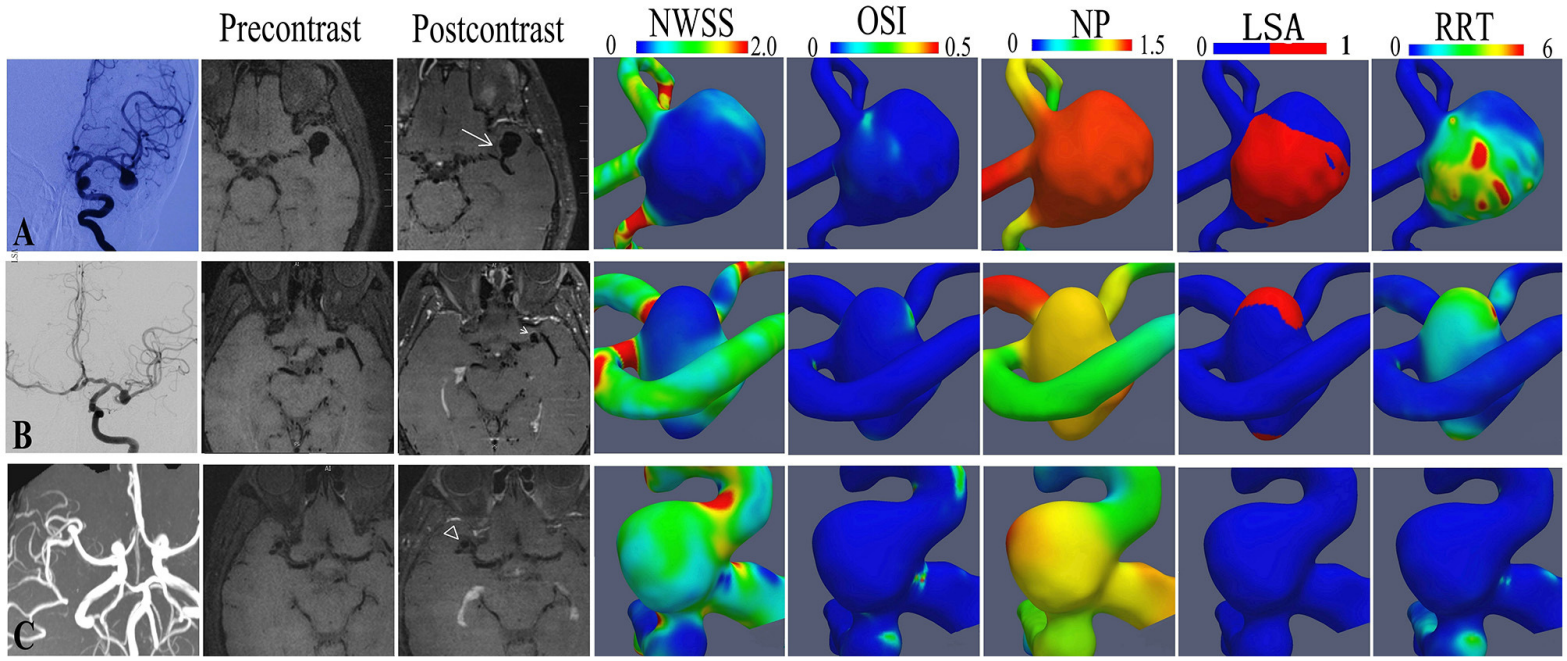
The hemodynamic features of representative aneurysms with different aneurysm wall enhancement patterns. A bifurcation aneurysm of the middle cerebral artery (MCA) with circumferential wall enhancement **(A)**, a bifurcation aneurysm of the middle cerebral artery (MCA) with partial wall enhancement **(B)**, and a bifurcation aneurysm of the MCA without enhancement **(C)**.

Aneurysm wall enhancement was assessed using a 3T MRI (Siemens, Munich, Germany). The aneurysm location was identified by 3D time-of-flight magnetic resonance angiography. Precontrast and postcontrast T1-weighted dark-blood fast spin-echo MRI sequence was then performed. The scanning parameters were as follows: repetition time, 700 ms; echo time, ms; echo train length, 6; field of view, 130 mmx30 mm; acquired matrix, 256x256; slice thickness, 2.0 mm; total slab thickness, 4 cm; flip angle, 180°; and total imaging time, 5 min. Postcontrast T1-weighted MRI was performed approximately 1 min after intravenous injection of Gd-DTPA (Magnevist, Bayer, Germany) at a dose of 0.1 mmol/kg. This study defined AWE as an aneurysm wall with a signal intensity equal to or greater than that of the pituitary stalk on a postcontrast T1-weighted MRI scan that was not present on MRI before gadolinium administration. The AWE patterns of each aneurysm were classified into three groups: no AWE, partial AWE (part of the wall was enhanced, and the rest was slightly enhanced or not enhanced), and circumferential AWE (almost the whole wall was homogeneously enhanced) (Figure 1). Two experienced neuroradiologists reviewed the MRI scans independently and determined whether AWE was present or not; discordances were resolved by discussion.

### Statistical Analysis

Continuous variables were expressed as median (interquartile range), and categorical variables were expressed as frequency and percentage. The Shapiro–Wilk test was used to determine the distribution characteristic. The chi-square test or the Kruskal–Wallis H test investigated the differences in clinical baseline and hemodynamic data between AWE patterns. The significant hemodynamic variables in a univariate analysis were then added into an ordinal logistic regression to identify independent risk factors for AWE patterns. Pearson's correlation was also used to assess the correlation coefficient between hemodynamic variables and AWE patterns. *P*-values <0.05 were considered to indicate the statistical significance and P-values between 0.05 and 0.10 were considered to indicate a trend. All the data were analyzed with SPSS 20.0 (IBM, Armonk, New York).

## Results

### Clinical Characteristics

A total of 28 patients with 32 saccular bifurcation aneurysms of the MCA were included in this study. Microsurgery was performed on 13 aneurysms and embolization on 18 aneurysms while one aneurysm in a patient with bilateral MCA aneurysm was managed conservatively. The sample consisted of 9 men (32.1%) and 19 women (67.9%), with a median age of 56 years (range 45–72 years). Four cases (14.3%) had bilateral MCA aneurysms. The median aneurysm size was 5.26 mm (ranging from 3.15–22.6 mm), where 15 aneurysms (46.9%) were irregular, and 17 (53.1%) were located on the right side. Thirteen aneurysms (40.6%) showed AWE, and 19 (59.4%) showed non-enhancement on vessel wall MRI scan. Among the aneurysms with AWE, seven aneurysms (53.8%) showed partial AWE, and six (46.2%) showed circumferential AWE. A 3D-DSA was the first choice for hemodynamic analysis; if 3D-DSA was unavailable, computed tomography angiography was used. Computational fluid dynamic data were generated from preoperative 3D-DSA in 26 aneurysms and computed tomography angiography data in 6 aneurysms.

**Figure 2:**
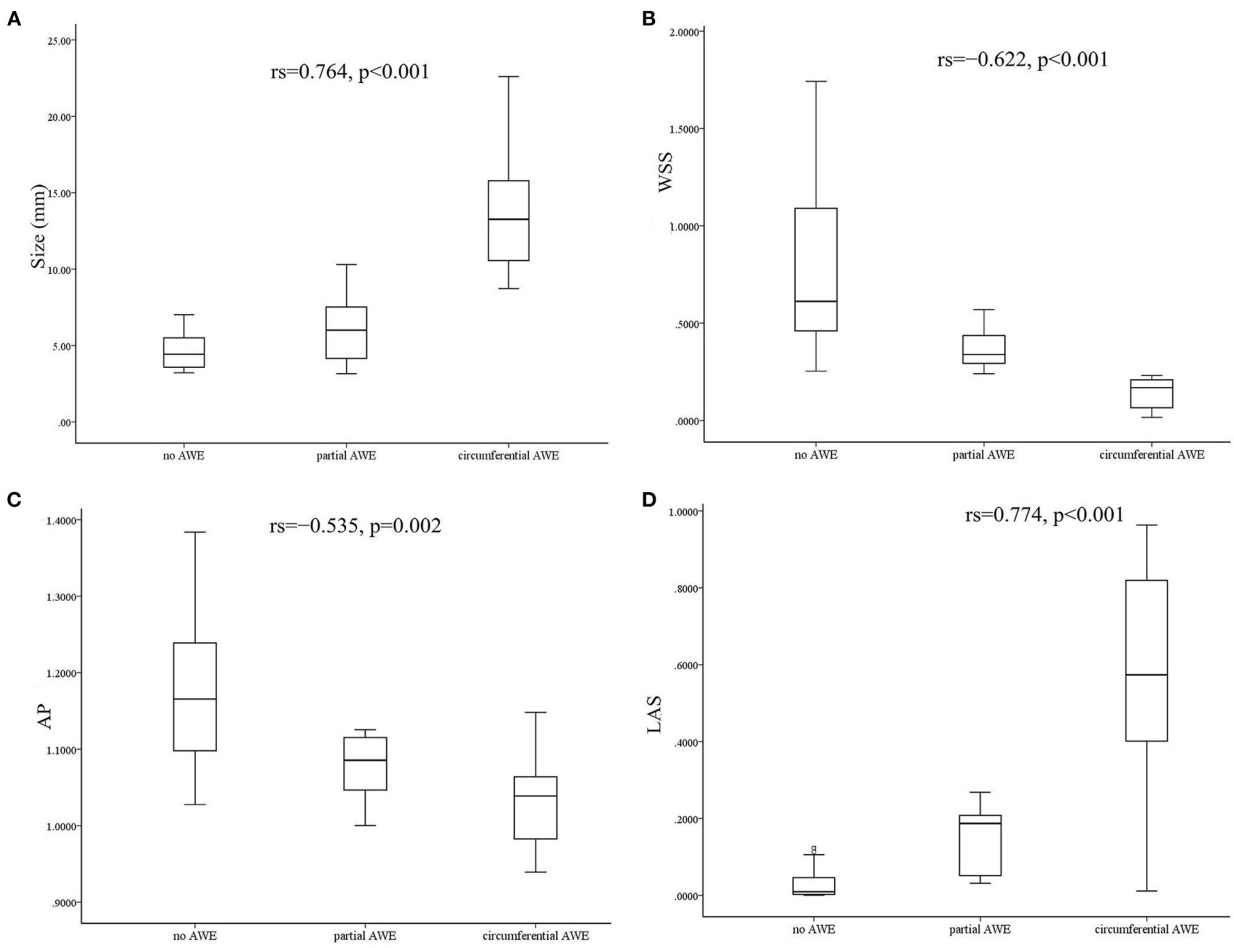
Box diagram and Spearman's correlation analysis showed that aneurysm size was positively correlated with aneurysm wall enhancement (AWE) **(A)**. Wall shear stress (WSS) was negatively correlated with AWE **(B)**. Aneurysm pressure (AP) was negatively correlated with AWE **(C)**, and low shear area (LSA) was positively correlated with AWE **(D)**.

Demographic features (sex and age), medical history (hyperlipidemia, hypertension, and diabetes), and risk factors (smoking and drinking) were not associated with AWE patterns (*P* > 0.05) in this study (Table 1). Aneurysm irregularity was also not associated with AWE patterns. The size of aneurysms was significantly associated with AWE in univariate analysis. The median size of aneurysms was 13.26 mm (range 8.73–22.6 mm) for circumferential AWE, 6.0 mm (range 3.15–10.3 mm) for partial AWE, and 4.43 mm (range 3.21–7.01 mm) for aneurysms with non-enhancement on vessel wall MRI scan. Spearman's correlation analysis showed that the aneurysm size was positively correlated with the AWE pattern (rs = 0.764, *p* < 0.001) (Figure 2). The size of the aneurysm was also positively associated with RRT (rs = 0.757, *p* < 0.001) and LSA (rs = 0.854, *p* < 0.001) but negatively correlated with WSS (rs = −0.528, *p* = 0.002) and AP (rs=-0.380, *p* = 0.032).

**Table 1:** The clinical characteristics of unruptured intracranial aneurysms with different aneurysm wall enhancement (AWE) patterns.

	**No AWE(n = 19)**	**partial AWE(n = 7)**	**circumferential AWE(n = 6)**	***P*-value**
Age	56 (53–66)	56 (51–64)	57 (52–68)	0.973
Female	14 (43.8)	5 (15.6)	3 (9.4)	0.543
Hypertension	15 (46.9)	6 (18.8)	3 (9.4)	0.274
Diabetes	6 (18.8)	0 (0.0)	0 (0.0)	0.080
Hyperlipidemia	2 (6.3)	0 (0.0)	0 (0.0)	0.482
Current drinking	0 (0.0)	1 (3.1)	0 (0.0)	0.158
Current smoking	3 (9.4)	2 (6.3)	0 (0.0)	0.368
Cholesterol (mmol/L)	3.17 (2.67–4.57)	4.69 (2.56–4.98)	3.73 (2.83–4.16)	0.608
Triglyceride (mmol/L)	1.20 (0.91–1.49)	1.4 (1.08–2.12)	0.97 (0.69–1.88)	0.259
High-density lipoprotein (mmol/L)	1.20 (0.87–1.3)	1.11 (0.75–1.22)	1.04 (0.82–1.39)	0.600
Low-density lipoprotein (mmol/L)	1.68 (1.30–2.94)	1.86 (1.42–3.37)	1.64 (1.42–1.89)	0.607
Irregular aneurysm	9 (28.1)	4 (12.5)	2 (6.3)	0.691
Size (mm)	4.43 (3.30–5.70)	6.0 (3.78–7.80)	13.26 (10.10–17.49)	0.001

*Variables are expressed as median (interquartile range) or number of patients (%)*.

### The Hemodynamic Variables Associated With AWE

The associations between hemodynamic variables and AWE patterns are shown in Table 2. Variables such as WSS, OSI, AP, RRT, and LSA were significantly associated with AWE patterns in the Kruskal–Wallis H test analysis (*p* < 0.005). The significant variables in the univariate analysis were then added into an ordinal logistic regression to identify the independent risk factors for AWE patterns. The ordinal logistic regression analysis found that WSS was the only factor significantly associated with the AWE pattern (*p* = 0.048); a similar trend was observed for AP (*p* = 0.058) and LSA (*p* = 0.055), respectively. Spearman's correlation analysis was then used to assess the correlation coefficient between the independent hemodynamic variables and AWE patterns. Wall shear stress (rs = −0.622, *p* < 0.001) and AP (rs = −0.535, *p* = 0.002) were negatively correlated with AWE, while LSA was positively correlated (rs = 0.774, *p* < 0.001) (Figure 2).

**Table 2:** The hemodynamic characteristics of unruptured intracranial aneurysms with different aneurysm wall enhancement (AWE) patterns.

	**AWE pattern**			**Kruskal–Wallis H test**	**Multivariate analysis**
	**No AWE (*n* = 19)**	**Partial AWE (*n* = 7)**	**Circumferential AWE (*n* = 6)**	**P-value**	**P-value**
WSS	0.6118 (0.4500–1.1685)	0.3388 (0.2559–0.4911)	0.1686 (0.0529–0.2146)	<0.001	0.048
OSI	0.0068 (0.0048–0.0125)	0.0189 (0.0096-0.0206)	0.0148 (0.0107–0.0282)	0.017	0.172
AP	1.1655 (1.0969–1.2475)	1.0855 (1.0450–1.1193)	1.0388 (0.9719–1.0849)	0.011	0.058
RRT	0.0610 (0.0231–0.1031)	0.2761 (0.0946–0.8397)	1.1566 (0.3829–10.3336)	0.002	0.132
LSA	0.0096 (0.0019–0.0508)	0.1872 (0.0319–0.2140)	0.5736 (0.3038–0.8555)	<0.001	0.055

*Variables are expressed as median (interquartile range)*.

*WSS, wall shear stress; OSI, oscillatory shear index; AP, aneurysm pressure; RRT, relative residence time; LSA, low shear area*.

## Discussion

Our study found that low wall shear stress was associated with aneurysm wall enhancement on vessel wall MRI. Low aneurysm pressure and increased low shear area tended to be associated with AWE, although oscillatory shear index (OSI) was not associated with AWE. Previous reports also found that AWE aneurysms had lower WSS than non-AWE aneurysms (14, 15, 18) and that the enhanced area had lower WSS than the non-enhanced area in the same aneurysm (13, 16, 17).

Physiological WSS is important to maintain vascular endothelial cell survival and integrity. Previous animal studies have found that high WSS contributed to aneurysm formation and development (2). After formation, the high WSS in the aneurismal region may change into a low WSS region as the aneurysm enlarges with a corresponding increase in the low WSS area (24). Low WSS induces endothelial apoptosis and increases the endothelial permeability (24), and the damaged endothelial cell may trigger proinflammatory cytokines, such as IL-6, and adhesion molecules, such as MCP-1, inducing inflammatory cell infiltration (25, 26). Meanwhile, aneurysm flow velocity reduces, and the stagnation in aneurysms further facilitates inflammatory cell infiltration, lipid infiltration, atherosclerosis, and thrombosis formation (27). Inflammatory cell infiltration plays an important role in atherosclerosis. Intraluminal thrombus promotes inflammatory cell accumulation and microvascularization of the aneurysm wall (28). The repeated microbleeds from these new fragile vasa vasorum further promote inflammatory cell accumulation (28). Acute and chronic inflammatory cell accumulations accelerate aneurysm enlargement and induce aneurysm rupture (29).

A recent animal study demonstrated that the growth regions of the aneurysm were often exposed to low WSS and high OSI areas. Macrophage infiltration was also colocalized with the growing regions of the aneurysm (4). Clinical studies, similarly, found that the aneurysm growth region mainly occurred in aneurysm areas with low WSS and higher OSI (5, 6). A meta-analysis also found that low WSS was an important predictor of IA rupture (30). However, evidence remains conflicting. Takao et al. found no association between aneurysm rupture status and low WSS (31), while Cebral et al. reported that ruptured aneurysms were more likely to have high WSS (32). Low WSS has also been associated with the thin region of the IA wall in some studies (33, 34). However, several studies found that thick aneurysm walls with inflammatory cell infiltration and atherosclerosis were negatively correlated with WSS and positively correlated with RRT (7, 27, 35). For instance, Meng et al. postulated that high and low WSS might drive intracranial aneurysm growth and rupture through different pathological mechanisms, with low WSS facilitating inflammatory cell infiltration (36).

Previous studies have reported that aneurysm wall thickening accompanied by atherosclerosis, neovascularization, and intraluminal thrombus were associated with AWE (8–10). The above pathological changes are often accompanied by inflammatory cell infiltration, so AWE on vessel wall MRI may be an indirect marker of aneurysm wall inflammation. Low WSS may facilitate inflammatory cell infiltration and stimulate pathological changes such as atherosclerosis and intraluminal thrombus within the aneurysm wall. Furthermore, due to the increased vascular permeability and the slow blood flow, the passive exudation of contrast medium to aneurysm wall occurs more easily. This may explain the association between low WSS and AWE reported in the literature, and AWE may also indicate an unstable aneurysm with low WSS.

The association between AWE and other hemodynamic variables, such as RRT, LSA, OSI, and AP, remains controversial. Previous studies found that hyperplastic aneurysm regions had lower AP while thin aneurysm regions had elevated AP (33, 35). Spearman's correlation analysis found that AP was positively associated with WSS in this study, and low AP was associated with AWE. Increased LSA indicated that a high percentage of the aneurysm wall was exposed to the low WSS area, so increased LSA was also associated with AWE in our study, consistent with previous reports (16). Some studies found that the aneurysm wall enhancement region had a lower OSI (13, 16, 17). However, we did not observe this, although this may be due to different research methods and aneurysm selection criteria used in this study. WSS was also negatively correlated with RRT in this study; longer RRT may indicate low WSS with lower flow velocity and even stagnation of blood flow within the aneurysm, which would facilitate inflammatory cell infiltration and contrast agent exudation. However, the multivariate analysis did not reveal an association between RRT and AWE in this study. The size of the aneurysm was also positively associated with AWE (10, 37). In our study, a large aneurysm was usually related to low WSS, long RRT, or increased LSA, facilitating inflammatory cell infiltration and contrast agent exudation, thereby causing aneurysm wall enhancement.

The lack of correlation between hemodynamic parameters and aneurysm wall enhancement has been previously reported (19). The complexity and heterogeneity of the aneurysms included in the studies may explain these conflicting results regarding hemodynamics and aneurysm remodeling. There is considerable variation in the morphological characteristics of aneurysms, including aneurysm size, irregularity, and pathological features such as wall thickness, atherosclerosis, and inflammatory cell infiltration. Cerebral blood flow (CBF) findings can only register hemodynamic parameters of an aneurysm at a particular time. The aneurysm remodeling process is complex and may still be ongoing before rupture, so the morphology and hemodynamics within the aneurysm may vary accordingly (38). Additionally, aneurysm rupture itself could change hemodynamics, and studies comparing ruptured and unruptured aneurysms should be interpreted with caution. It is also essential to distinguish between aneurysm formation and growth. These processes have distinct hemodynamic features, so aneurysms of size <3 mm have been excluded in this study, which may influence our hemodynamic results. Whether our study results could be applied to aneurysms of any size deserve further investigation. Furthermore, differences between previous studies in hemodynamic parameter definition, methodology, and analysis would also lead to heterogeneous results, so the association between hemodynamics and AWE requires further investigation.

This study has some limitations. First, this was a single-center study with a small sample size, and only bifurcation aneurysms of the middle cerebral artery were investigated. The size and shape of the included aneurysms also varied. Therefore, selection bias cannot be excluded. Second, since most included aneurysms were treated at the time of diagnosis of the aneurysm, it was not confirmed which of the included aneurysms were unstable in the future. Our study only investigated the association between abnormal hemodynamics and AWE in unruptured aneurysms. Therefore, this study did not examine the association between AWE, hemodynamic, and aneurysm instability, such as growth and rupture. Future longitudinal studies with a large sample size and sufficient follow-up evaluations are needed. Furthermore, the following factors may have caused errors in the hemodynamic results: this study used a standardized input condition as patient-specific flow conditions were not available; arterial walls were assumed as rigid vessel walls, and the blood was assumed as a Newtonian fluid; and physiologic pulsatile flow conditions from healthy subjects were used. Hemodynamic analysis was not all generated from DSA or CTA data in this study; different imaging techniques may cause inconsistent hemodynamic results, which may influence our hemodynamic results. Meanwhile, sac-averaged hemodynamic factors were used in the analysis. Local hemodynamics factors related to the AWE area in one aneurysm were not analyzed, which may have resulted in some information loss. To our knowledge, AWE in some large aneurysms may result from slow intraaneurysmal flow along the wall rather than actual enhancement (39); hence, the finding of AWE in larger aneurysms should be carefully interpreted. Meanwhile, the resolution of 3.0-T MRI may not be sufficient for small aneurysms or aneurysms with very thin walls. The AWE could have been a false negative because of the partial volume effect in this study, which also influences our results. The location of the aneurysm could influence hemodynamic variables and AWE within the aneurysm. As our study was limited to investigating MCA aneurysms, further research may be required to determine whether our study results could be applied to aneurysms in other locations. The methods of assessing AWE are heterogeneous and have not been standardized in the literature. A simple qualitative grading is used in our study to evaluate AWE; however, an objective and quantitative assessment of wall enhancement may be more reliable. Aneurysm wall enhancement has been proposed as an indirect marker of aneurysm wall inflammation. However, AWE is not specific for inflammation, atherosclerosis, or neovascularization, as intraluminal thrombus within the aneurysm wall could also be enhanced. In our study, AWE was not evaluated by intraoperative inspection or histological assessment. Therefore, the pathological mechanisms for their association with abnormal hemodynamics and AWE in unruptured aneurysms should be interpreted with caution and further studied. However, similar studies elucidating the interactions between abnormal hemodynamics and AWE are rare. Nevertheless, this study provides some valuable information and hemodynamic evidence for AWE on vessel wall MRI.

## Conclusion

Low wall shear stress, low aneurysm pressure, and increased low shear area were associated with aneurysm wall enhancement on vessel wall magnetic resonance imaging in unruptured cerebral aneurysms. These abnormal hemodynamic parameters may induce inflammation and cause aneurysm wall enhancement. However, the association between these parameters and the underlying pathological mechanism requires further investigation.

## Data Availability Statement

The raw data supporting the conclusions of this article will be made available by the authors, without undue reservation.

## Ethics Statement

The studies involving human participants were reviewed and approved by the Ethics Committee of Qilu Hospital of Shandong University. Written informed consent from the patients/participants or patients/participants' legal guardian/next of kin was not required to participate in this study in accordance with the national legislation and the institutional requirements.

## Author Contributions

WZ: article drafting and writing. YD and HK: data collection and statistics. ML: computational fluid dynamics analysis. FX and XB: aneurysm wall enhancement analysis. DW, WS, and YW: review this article. All authors contributed to the article and approved the submitted version.

## Funding

This study was sponsored by the National Natural Science Foundation of China (grant number: 81701160).

## Conflict of Interest

WZ was affiliated with Lunan Pharmaceutical Group Co. Ltd. The remaining authors declare that the research was conducted in the absence of any commercial or financial relationships that could be construed as a potential conflict of interest.

## Publisher's Note

All claims expressed in this article are solely those of the authors and do not necessarily represent those of their affiliated organizations, or those of the publisher, the editors and the reviewers. Any product that may be evaluated in this article, or claim that may be made by its manufacturer, is not guaranteed or endorsed by the publisher.
